# Hollow Hydrogel Microfiber Encapsulating Microorganisms for Mass-Cultivation in Open Systems

**DOI:** 10.3390/mi8060176

**Published:** 2017-06-03

**Authors:** Kazuhiko Higashi, Miho Ogawa, Kazuma Fujimoto, Hiroaki Onoe, Norihisa Miki

**Affiliations:** 1 School of Integrated Design Engineering, Keio University, 3-14-1 Hiyoshi, Kohoku-ku, Yokohama, Kanagawa 223-8522, Japan; kazuhiko@z2.keio.jp (K.H.); notuned.miho@gmail.com (M.O.); fkazuma2007@gmail.com (K.F.); onoe@mech.keio.ac.jp (H.O.); 2 Department of Mechanical Engineering, Keio University, 3-14-1 Hiyoshi, Kohoku-ku, Yokohama, Kanagawa 223-8522, Japan

**Keywords:** microorganisms, mass-cultivation, contamination, open cultivation system, hydrogel, microfluidics

## Abstract

Open cultivation systems to monoculture microorganisms are promising for the commercialization of low-value commodities because they reduce the cultivation cost. However, contamination from biological pollutants frequently impedes the process. Here we propose a cultivation method using hollow hydrogel microfibers encapsulating microorganisms. Due to the pore size, hydrogels allow nutrients and waste to pass through while preventing invading microorganisms from entering the microfiber. Experimental cultivation shows the growth of target bacteria inside the alginate hydrogel microfiber during exposure to invading bacteria. The membrane thickness of the microfiber greatly affects the bacterial growth due to changes in membrane permeability. The enhancement of mechanical toughness is also demonstrated by employing a double-network hydrogel for long-term cultivation. The hollow hydrogel microfiber has the potential to become a mainstream solution for mass-cultivation of microorganisms in an open system.

## 1. Introduction

Microorganisms, which utilize complex metabolic reactions made increasingly sophisticated by natural selection, have been powerful and useful chemical plants throughout human history. Due to the fast growth rate of microorganisms and the availability of inexpensive culture medium, their diverse metabolites have been used in various industries [1], including agriculture, food, wastewater treatment, pharmaceuticals, and biofuel. With the emergence of recombinant DNA technology, even eukaryotic biomolecules for therapeutics (e.g., insulin [2], human growth hormone [3]) and structural materials (e.g., spider silk [4], resilin [5]) have been produced in a microbial body.

To maximize the cost-effectiveness of white biotechnology, the methodology of microbial cultivation is critically important. Regarding mass-cultivation of microorganisms on an industrial scale, the cultivation systems are generally classified as closed or open [6]. The advantages of the closed cultivation system include good control of the cultivation condition and a reduced risk of contamination. However, the complex and expensive facility of the system increases the cost of the final product. On the other hand, an open cultivation system is cost-effective because it has fewer equipment requirements and can potentially be scaled up. Especially for the production of low-value commodities such as biofuels by photosynthetic microorganisms, cost is the most important dominant factor [7]. Therefore, an open cultivation system is the only choice for the cultivation of photosynthetic microorganisms [8].

For open cultivation systems, contamination from invading heterotrophs, pathogens, and grazers, as well as dust and sand, is the main restriction for commercial viability since the contaminants greatly reduce the growth rate of the target microorganisms [9]. Thus, many researchers have proposed open cultivation methods to overcome the problem of animate and inanimate contamination. One approach is to use chemical additives such as quinine sulfate [10,11], rotenone [12], and ammonium hydroxide [13]. Yet this method decreases the cell growth rate. Another approach is to use selective environments, such as high salinity or alkalinity [14]. However, this approach constrains the usable species (e.g., *Dunaliella salina* or *Spirulina*). The other solution to avoid the risk of contamination is to control the process for the desired culture condition, such as a temporary reduction in pH, thereby enabling a fast growth rate of the target microbes [15]. Although this strategy is applicable to various species, the additional costs necessary to install and operate a system for controlling the environment are prohibitive.

**Figure 1:**
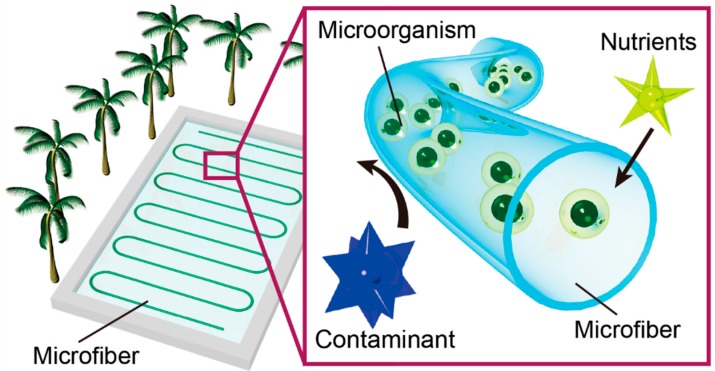
Conceptual schematic of the hollow hydrogel microfiber containing microorganisms in an open cultivation system. The pore size of the hydrogel allows nutrients to pass through the fiber but prevents contaminants from entering the fiber.

We previously introduced a concept for a new cultivation technique using hollow hydrogel microfibers to cultivate microorganisms in open cultivation systems [16]. In the present study, we conduct detailed analyses of the method, including enhancement of the mechanical toughness utilizing a double-network hydrogel. Figure 1 shows a conceptual schematic of the method; hollow hydrogel microfibers encapsulating the target microorganisms, which are produced using a three-dimensional co-axial microfluidic device, are immersed into open environments such as lakes and ponds. The pore size of the hydrogel fiber membrane allows nutrients, gases, and waste to pass through while preventing invading heterotrophs and viruses from entering the fiber [17]. This simple approach significantly reduces costs by using alginate, which is an earth-abundant biopolymer, and employing a microfluidic fabrication process, which is easy for scale-up and automation. In this study, we demonstrate cultivation of *Corynebacterium glutamicum* as the target microbe encapsulated in the microfiber for the proof of concept. First, the hollow hydrogel microfiber is formed by using a co-flowing microfluidic device. Second, growth rates of *C. glutamicum* with and without the hydrogel fiber are compared, employing *Vibrio alginolyticus* as an invading heterotroph. Third, the effect of the fiber wall thickness on the microbial growth rate is evaluated for characterization of the system. Finally, the applicability of the double-network hydrogel to increase the durability of the microfiber is assessed.

## 2. Experimental Section

### 2.1. Materials and Reagents

*C. glutamicum* (NBRC 12168) and *V. alginolyticus* (NBRC 15630) were used as the target bacterium and invading bacterium, respectively. The chemicals for cultivating the bacteria, including MgSO_4_·7H_2_O, ethanol, polypepton, yeast extract, and Daigo’s Artificial Seawater SP for Marine Microalgae Medium, were purchased from Wako Pure Chemical Industries, Japan. Deionized (DI) water was prepared using a Millipore system (Direct-Q3, Millipore, Bellerica, MA, USA).

For the materials to form the hollow hydrogel microfibers, sodium alginate (80–120 cP), and calcium chloride were purchased from Wako Pure Chemical Industries, Japan. For the double-network hydrogel, 30% w/v-Acrylamide/Bis Mixed Solution (29:1), ammonium peroxodisulfate (APS), and *N*,*N*,*N*′,*N*′-Tetramethylethylenediamine (TEMED) were purchased from Nacalai Tesque, Inc., Kyoto, Japan.

Blue fluorescent microspheres (F8814, 1.0 μm) and green fluorescent microspheres (G0100, 1.0 μm) for fluorescent imaging of the produced hollow microfibers were purchased from Thermo Fisher Scientific Inc., Waltham , MA, USA.

### 2.2. Microfluidic Formation of Hollow Hydrogel Microfiber

**Figure 2:**
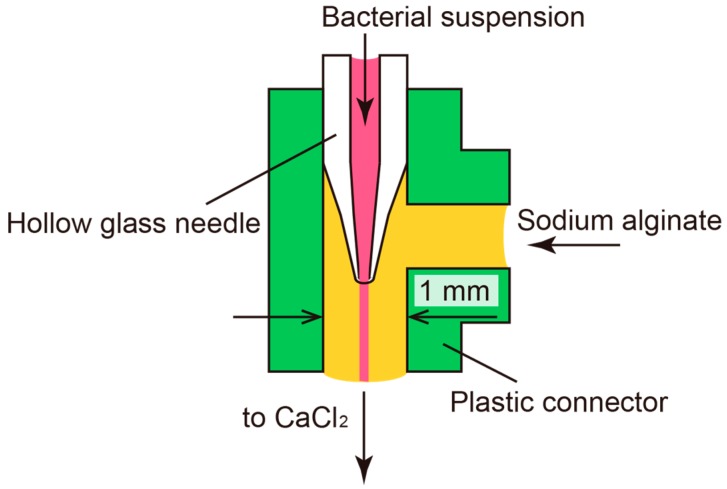
Formation of the microfiber using a co-flowing microfluidic device. The bacterial suspension is introduced from a hollow glass needle with an inner diameter of 100 μm at the tip. Sodium alginate solution is introduced from the other inlet. The produced coaxial laminar flow is extruded into a calcium chloride solution, which results in the formation of a hollow microfiber made of calcium alginate hydrogel.

Figure 2 shows the co-flowing microfluidic device comprising a plastic connector (VTF 106, AS ONE, Osaka, Japan) with an inner diameter of 1.3 mm and a hollow glass needle. The design of the device is similar to designs reported elsewhere [18,19,20]. The glass needle was formed by pulling a hollow glass tube (Hirschmann Laboratory, Eberstadt, Germany) with an inner diameter of 0.47 mm and an outer diameter of 1.2 mm to have a sharp edge at the tip using a Puller PC-10 (Narishige Group, Tokyo, Japan). A coaxial laminar flow was formed by introducing a bacterial suspension from the inlet of the glass needle and sodium alginate solution (1.5% w/v) from the other inlet. The sodium alginate solidifies at the outlet when it encounters Ca^2+^ ions in a 150 mM calcium chloride solution. Thus, a hollow hydrogel microfiber encapsulating bacteria was obtained. The diameter of the microfiber can be controlled by changing the flow rates of the inner and outer flow. Syringe pumps (AS ONE) were used in the present study to control the flow rates.

### 2.3. Culture of Bacteria in Hollow Microfiber

As the proof-of-concept experiment, *C. glutamicum* was used as the target bacterium. This bacterium has an elliptical shape and produces lactic acid. First, we measured the bacterial growth rates with and without the microfiber. The microfiber containing *C. glutamicum* was immersed into the culture medium, and the cell density was measured every 3 h. The cell density in the microfiber was measured by visually counting the number of *C. glutamicum* extracted from the fiber by pipetting. For the control condition, *C. glutamicum* was cultivated in bulk culture medium composed of 10 g of polypepton, 2 g of yeast extract, 1 g of MgSO_4_·7H_2_O, and 1 L of DI water.

Next, we tested whether the microfiber can prevent other bacteria from entering the microfiber. As a control experiment, we cultured *C. glutamicum* in bulk culture medium containing *V. alginolyticus*. Then, we cultivated *C. glutamicum* in the microfiber in culture medium containing *V. alginolyticus*. The cell densities of the bacteria for both conditions were measured for 1 h. *V. alginolyticus* is motile, making it easy to distinguish both bacteria. Additionally, the effect of the membrane thickness of the microfiber on the cell growth rate was investigated by measuring the concentration of lactic acid, which is a by-product of *C. glutamicum*, inside the microfiber by pipetting. The measurement was conducted 2 h after cultivation in 20 mL of culture medium using an F-kit (l-lactic acid, Roche Diagnostics, Basel, Switzerland) and an ultraviolet-visible-near-infrared spectrophotometer (UV-Vis-NIR; U-3310, Hitachi, Tokyo, Japan).

### 2.4. Double-Network Hollow Hydrogel Microfiber

A double-network hydrogel is a material composed of two different polymer chains—one with rigid chains, and the other with flexible chains—that exhibits extremely high mechanical toughness and stretchability [21]. When the gel is stretched, the rigid polymer chain ruptures and dissipates energy. Since the alginate hydrogel is brittle and biodegradable, a tougher material could be suitable for long-term cultivation. We assessed the applicability of using a double-network hydrogel composed of alginate and polyacrylamide [22] in our system.

To prepare the double-network hydrogel microfiber, a bacterial suspension of *C. glutamicum* was introduced into the glass needle, and sodium alginate solution mixed with acrylamide/bis solution and APS was introduced into the other inlet channel. The coaxial flow was extruded into the mixed solution of calcium chloride and TEMED, resulting in formation of the hollow microfiber of the double-network hydrogel. Double-network hydrogel microfibers with six different mix ratios were prepared. For all samples, concentrations of APS and TEMED were fixed at 5.1 mM and 8.6 mM, respectively.

To assess its mechanical properties, the microfiber was stretched using a tensile machine (RSAIII, TA Instruments, New Castle, DE, USA). The outer diameter, membrane thickness, and length of the fiber were fixed at 1.5 mm, 100 μm, and 10 mm, respectively. The rate of stretch was kept constant at 0.1 mm/s.

To investigate the effect of polyacrylamide in the double-network hydrogel on the metabolism of *C. glutamicum*, the double-network hydrogel microfiber was immersed in the 20 mL culture medium for 3 h. After the cultivation, the amount of lactic acid was measured using an F-kit and UV-Vis-NIR spectrophotometer. All the membrane thicknesses of the fiber were 100 μm.

## 3. Results and Discussion

### 3.1. Culture of Bacteria in Hollow Microfiber

**Figure 3:**
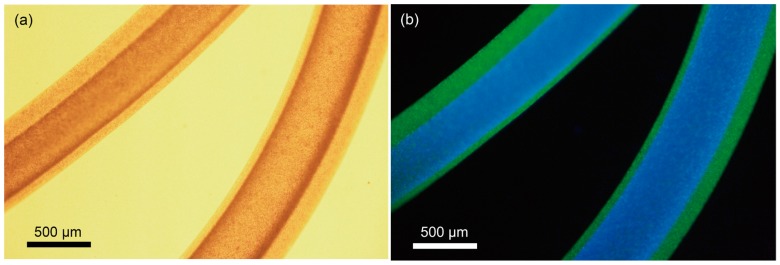
Micrographs of the hollow hydrogel microfiber. (**a**) Optical micrograph. (**b**) Fluorescent micrograph corresponding to (a). The fiber is colored with blue and green fluorescent particles for the core and the shell region, respectively.

**Figure 4:**
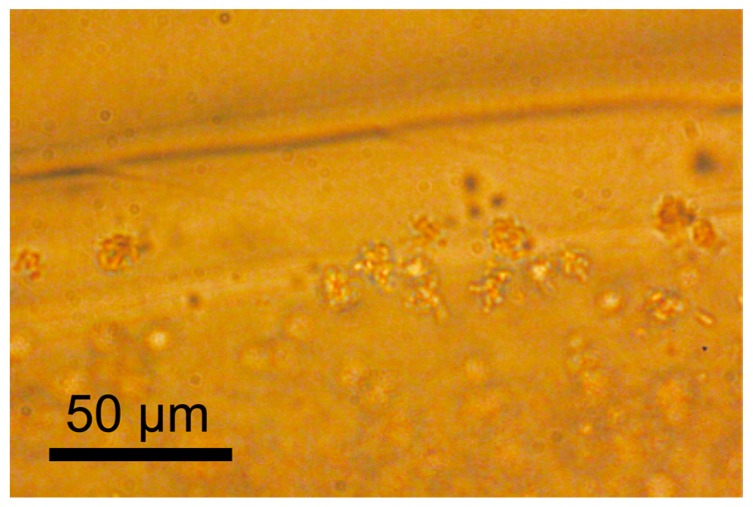
Micrograph showing *C. glutamicum* in the microfiber.

**Figure 5:**
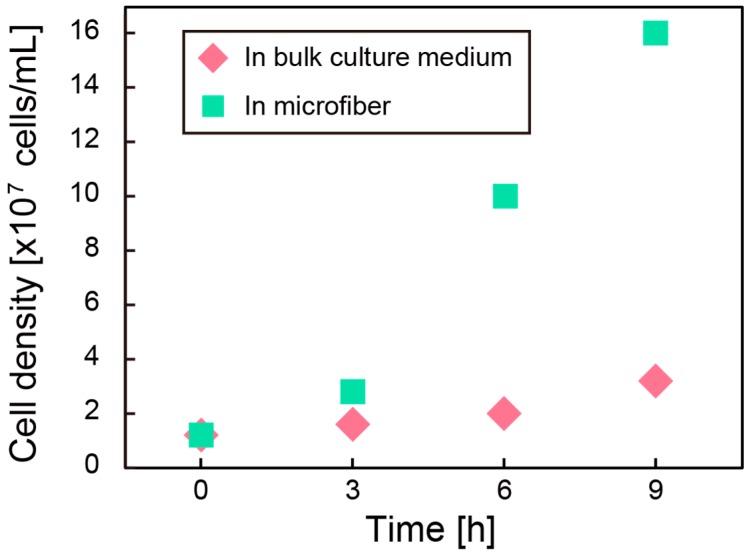
Cell density of *C. glutamicum* as a function of culture time. The measurements were conducted every 3 h for both conditions.

Figure 3 shows a micrograph of the hydrogel microfiber colored with fluorescent particles. This is a merged image of blue particles for the core region and green particles for the shell region, indicating the formation of the hollow hydrogel microfiber. The hollow microfiber encapsulating *C. glutamicum* was successfully formed, as shown in Figure 4. Figure 5 shows the cell density after cultivation in the bulk culture medium and the microfiber. The growth rate of the bacteria in the microfiber was faster than that without the microfiber. This is probably because the bacteria inside the microfiber were surrounded by sufficient bulk culture, enabling rapid exchange of oxygen, nutrients, and waste through the microfiber. In cases when the target microbes, such as *Aurantiochytrium*, internally store their by-products, it is necessary to extract the bacteria from the fiber. After cultivation for 9 h, the microfiber was dissolved with NaHCO_3_. Fluorescent observation using a green fluorescent nucleic acid stain (SYTO9) revealed that more than 80% of the bacteria remained alive.

**Figure 6:**
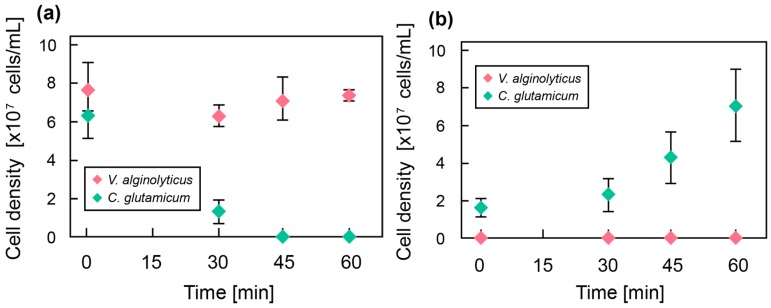
(**a**) Cell density of *C. glutamicum* and *V. alginolyticus* without the microfibers as a function of culture time. (**b**) Cell density of *C. glutamicum* in the microfiber exposed to a suspension containing *V. alginolyticus* as a function of culture time.

Next, we co-cultured *C. glutamicum* and *V. alginolyticus* with and without the microfiber. Figure 6 shows the cell densities of the bacteria (a) in a bulk culture medium and (b) inside the microfiber during cultivation for 1 h. Without the microfiber, all *C. glutamicum* were dead in 45 min. On the other hand, *C. glutamicum* in the hydrogel microfiber grew exponentially while the cell density of *V. alginolyticus* remained at zero, indicating that there was no invasion of *V. alginolyticus* into the microfiber. Based on these results, the microfiber could successfully protect the target bacteria from invading bacteria.

**Figure 7:**
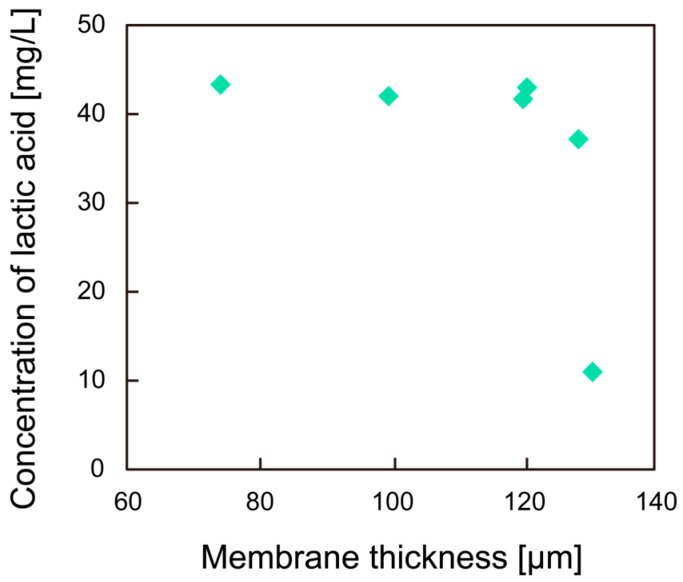
Concentration of the lactic acid produced by *C. glutamicum* as a function of the membrane thickness of the microfiber.

The effect of membrane thickness of the microfiber on the bacterial growth rate was also evaluated (Figure 7). By controlling the flow rate, the desired membrane thickness can be achieved. However, it was found that the microfiber did not form when the ratio of the volumetric flow rate of the sodium alginate solution to the bacterial suspension exceeded 50%. Therefore, microfibers with different membrane thicknesses were prepared at ratios of the volumetric flow rate less than 50%. As shown in Figure 7, lactic acid production sharply decreased when the membrane thickness was greater than 120 μm. This is probably because the nutrient supply rate by diffusion through the hydrogel membrane decreased with the thicker membrane, thereby restricting bacterial growth. Therefore, it is preferable that the membrane thickness of the microfiber is less than 120 μm.

### 3.2. Double-Network Hollow Hydrogel Microfiber

**Figure 8:**
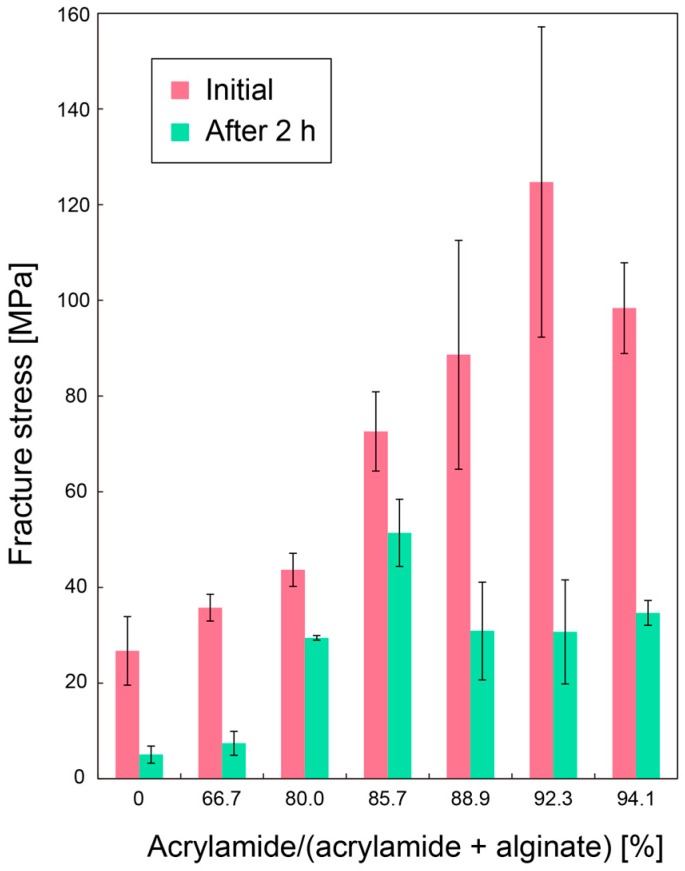
Fracture stress of the microfiber due to elongation as a function of the composition ratio of acrylamide and alginate.

Figure 8 shows the fracture stress of the microfiber due to elongation as a function of the composition ratio of the double-network hydrogel microfiber. The mechanical strength of the fiber was enhanced when the ratio for the initial condition was approximately 92.3. This result is consistent with the result of previous work [22]. However, all the fracture stresses were decreased after cultivation of *C. glutamicum* for 2 h. This is probably because the Ca^2+^ ions crosslinking alginate polymer chains were metabolized by the *C. glutamicum*, thereby reverting the alginate gel to the sol state. Nevertheless, the microfibers with composition ratios of 80–94.1% exhibited increased fracture stresses compared with the microfibers composed only of alginate after cultivation due to the remaining polyacrylamide. Therefore, the double-network hydrogel could be suitable for our system, especially for long-term cultivation.

**Figure 9:**
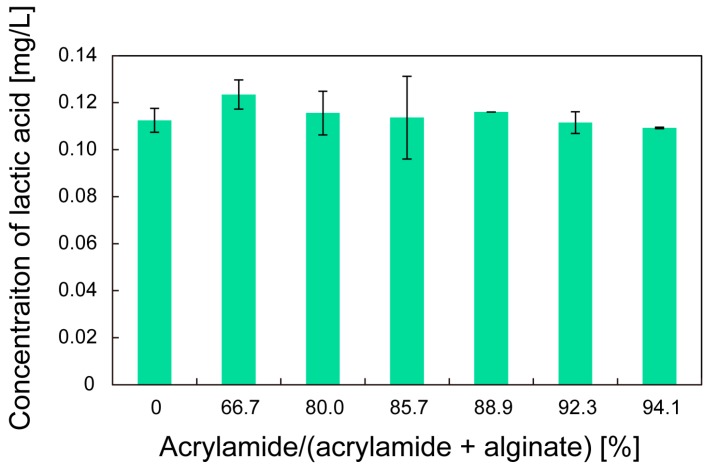
Concentration of the lactic acid produced by *C. glutamicum* as a function of the composition ratio of acrylamide and alginate after cultivation for 3 h.

Finally, the effect of the composition ratio on the growth rate of the bacteria was investigated. Figure 9 shows the concentration of lactic acid produced after cultivation for 3 h. For all the composition ratios, statistical analysis using analysis of variance did not show any significant differences. This indicates that the double-network hydrogels with different composition ratios had similar diffusion coefficients. Therefore, the double-network hydrogel has no effect on the cultivation of the bacteria.

## 4. Conclusions

We demonstrated a hollow hydrogel microfiber containing microorganisms for mass-cultivation in an open system. Microfibers with specific diameters and membrane thicknesses can be precisely formed using a simple microfluidic device. The target bacteria are cultivated in the microfiber while the invading bacteria are kept outside the microfiber. The membrane thickness of the microfiber greatly affects the growth rate of the bacteria; with the growth rate sharply decreasing at thicknesses exceeding 120 μm. However, a membrane that is too thin would reduce the mechanical toughness of the microfiber. The applicability of a double-network hydrogel to enhance the mechanical properties of the microfiber for our system was also assessed. The double-network hydrogel significantly increases the mechanical toughness of the microfiber without inhibiting bacterial growth. The hollow hydrogel microfiber has the potential to become a mainstream solution for mass-cultivation of microorganisms in an open cultivation system.
